# *Melanoseris zhufengensis* (Asteraceae, Cichorieae, Lactucinae), a new species from the Central Himalaya, Xizang, China

**DOI:** 10.3897/phytokeys.281.215554

**Published:** 2026-10-29

**Authors:** Li Yan, Yi Yang, Chun-Xia Ye, Ming Tang, Qing-Wen Sun, Ze-Huan Wang

**Affiliations:** 1 Department of Traditional Chinese Medicine Resources and Development, College of Pharmacy, Guizhou University of Traditional Chinese Medicine, Guiyang 550025, Guizhou, China Department of Traditional Chinese Medicine Resources and Development, College of Pharmacy, Guizhou University of Traditional Chinese Medicine Guiyang China; 2 Jiangxi Provincial Key Laboratory of Improved Variety Breeding and Efficient Utilization of Native Tree Species, Forestry College, Jiangxi Agricultural University, Nanchang 330045, China Jiangxi Provincial Key Laboratory of Improved Variety Breeding and Efficient Utilization of Native Tree Species, Forestry College, Jiangxi Agricultural University Nanchang China; 3 Jinjiang Yunyuan, Panlong District, Kunming, Yunnan 650224, China Unaffiliated Kunming China; 4 Guizhou Key Laboratory for Germplasm Innovation and Resource-Efficient Utilization of Dao-di Herbs, Guizhou University of Traditional Chinese Medicine, Guiyang 550025, Guizhou, China Guizhou Key Laboratory for Germplasm Innovation and Resource-Efficient Utilization of Dao-di Herbs, Guizhou University of Traditional Chinese Medicine Guiyang China

**Keywords:** Morphology, phylogeny, southern Xizang, taxonomy

## Abstract

*Melanoseris zhufengensis***sp. nov**., a new species from the Central Himalaya, Xizang, China, is described and illustrated. Similar to *M. violifolia* and *M. rapunculoides* in having five florets per capitulum, it can be distinguished by its caespitose habit, basally profusely branched stems, sessile middle cauline leaves with broad, unconstricted basal wings, phyllaries abaxially moderately white-pilose on the midvein, and achenes with three markedly broadened lateral ribs per surface and a 3.1–3.8 mm long beak. Integrative morphological comparisons and multi-locus phylogenetic analyses based on nuclear ITS and four plastid markers (*petD*, *trnL-F*, *rpl32-trnL*, *trnQ-rps16*) strongly support the recognition of *M. zhufengensis* as a distinct species of *Melanoseris*.

## Introduction

*Melanoseris* Decne. (Asteraceae, Cichorieae, Lactucinae) was originally established in 1844 to accommodate two Himalayan species (Decaisne 1844), but the genus name fell into disuse for more than 165 years until its reinstatement in the Flora of China treatment (Shih and Kilian 2011) based on early molecular phylogenetic results. Prior to this taxonomic revision, species now assigned to *Melanoseris* had long been scattered across multiple allied genera, including *Lactuca* L., *Cicerbita* Wallr., *Mulgedium* Cass., *Cephalorrhynchus* Boiss., *Prenanthes* L., *Chaetoseris* Shih, and *Stenoseris* Shih, leading to persistent confusion in species delimitation and interspecific relationships (Shih 1991, 1997; Zhu 2004; Zhu et al. 2004, 2006; Bano and Qaiser 2009, 2010; Abid et al. 2017; Ghafoor et al. 2017). Subsequent phylogenetic analyses using nrITS and multiple plastid DNA markers clarified the monophyly of the *Melanoseris* clade, confirming this genus as the most species-rich lineage of the subtribe Lactucinae within China (Wang et al. 2013, 2015; Kilian et al. 2017). Comprehensive taxonomic revisions across the Himalayas have documented roughly 30 recognized species distributed throughout the Pan-Himalayan biodiversity hotspot (Wang et al. 2013, 2015; Abid et al. 2017; Yin et al. 2018; Zhong et al. 2023; Xu et al. 2024).

*Melanoseris* exhibits extensive morphological variation and is prone to morphological plasticity, leading to frequent interspecific morphological overlap, which complicates species delimitation across its distribution range, particularly within Himalayan populations (Wang et al. 2013, 2015; Abid et al. 2017; Yin et al. 2018). Recent integrative taxonomic research combining morphological data and multi-locus molecular data has proven critical for resolving species taxonomy and documenting phylogenetic incongruence within *Melanoseris* (Wang et al. 2013; Kilian et al. 2017; Yin et al. 2018). To date, three new *Melanoseris* species have been formally published by our research team: *M. jilongensis* Ze H.Wang & H.Peng from southwestern Xizang near the Zhufeng (Qomolangma) National Nature Reserve (Yin et al. 2018), *M. kangdingensis* Ze H.Wang from western Sichuan (Zhong et al. 2023), and *M. penghuana* Ze H.Wang & H.J.Dong from north-central Yunnan (Xu et al. 2024). Among them, *M. jilongensis* represents a case of reticulate evolution and chloroplast capture that underpins the phylogenetic complexity within Himalayan *Melanoseris* lineages (Wang et al. 2013; Kilian et al. 2017; Yin et al. 2018).

Despite the progressive taxonomic revision of *Melanoseris* across the Pan-Himalayan region, some taxa still remain undiscovered or insufficiently characterized, especially in remote and botanically understudied alpine habitats of southern Xizang. During field surveys carried out in the alpine zones around Mount Qomolangma National Nature Reserve, a morphologically distinct population of *Melanoseris* was collected, which cannot be assigned to any previously described species of the genus based on vegetative and reproductive morphological characters, especially its distinctive capitulum and achene features.

In the present study, we perform comprehensive integrative taxonomic analyses for this putative new species. Morphological comparisons with *Melanoseris violifolia* and *M. rapunculoides*, the morphologically most similar congeners distributed in the Himalayas, are conducted, and scanning electron microscopy (SEM) is applied to examine the micromorphological sculpturing of the achenes in these three taxa. In addition, nrITS and four plastid gene fragments are amplified and sequenced to reconstruct phylogenetic trees, aiming to clarify its systematic position within *Melanoseris* and explore its evolutionary relationship among the Himalayan taxa.

The main objectives of this work are: (1) to formally describe and illustrate the new species of *Melanoseris* from the Qomolangma area; (2) to identify diagnostic features of the new species by morphological comparison with similar species; (3) to clarify the phylogenetic position of the new species and enrich our understanding of species diversity of *Melanoseris* in the Himalayan region.

## Materials and methods

### Morphological examination

Morphological characterization of the new species was performed based on *in situ* field observations and photographs of living individuals, supplemented by dried voucher specimens deposited at KUN and GZTM collected during our surveys. We studied taxonomic keys and species descriptions from Flora Reipublicae Popularis Sinicae (Shih 1997), Flora of China (Shih and Kilian 2011), and Flora of Pakistan (Abid et al. 2017), and physical specimens and digital images from the herbaria RAW, KUH, KUN, and PE for comparative analysis.

Features including ligule and anther tube length, achene dimensions, and pappus were observed and measured under an OD500H stereoscopic microscope. For micromorphological comparison of mature achenes between the new species and its morphologically closest allies, intact mature achenes were affixed to metallic stubs with double-sided adhesive tape and gold-coated before SEM observation. All morphological plates were edited and assembled using Adobe Illustrator CS4; achene micromorphological terminology follows Zhu et al. (2006).

### Molecular phylogenetic analyses

For molecular phylogenetic analysis, silica-dried leaves of two individuals of the new species were newly collected, and two leaf fragments of *Melanoseris rapunculoides* were sourced from KUH herbarium sheets. The nrITS and four plastid loci (*petD*, *trnL-F*, *rpl32-trnL*, *trnQ-rps16*) were amplified and sequenced following the protocols of Wang et al. (2013). All newly generated sequences from this study were deposited in INSDC (GenBank), with sample details and correspon­ding accession numbers for each locus listed in Table 1. We built separate nrITS and combined plastid datasets by integrating our new sequences with published data of *Melanoseris* and consistent outgroup taxa from Wang et al. (2013) and Kilian et al. (2017). The final nrITS matrix comprised sequences from 43 ingroup and four outgroup taxa, while the combined matrix of the four plastid loci contained sequences from 37 ingroup and the same four outgroup taxa.

**Table 1.** Information of newly sampled specimens.

**Taxon**	**Voucher (collector & number, herbarium)**	**Locality**	**GenBank accession**				
			**nrITS**	**petD**	**trnL-F**	**rpl32-trnL**	**trnQ-rps16**
*Melanoseris zhufengensis*-1	Ye Chunxia 20230801 (KUN)	Dingri, Xizang, China	PZ938932	PZ959010	PZ939615	PZ939619	PZ939623
*Melanoseris zhufengensis*-2	Tang Ming et al. JXAU-36 (GZTM)	Dingri, Xizang, China	PZ938933	PZ959011	PZ939616	PZ939620	PZ939624
*Melanoseris rapunculoides*-1	San Alam & Naeem Khan s.n. (KUH)	Gilgit, Gilgit-Baltistan, Pakistan	PZ938934	PZ959012	PZ939617	PZ93962	PZ939625
*Melanoseris rapunculoides*-2	Ali Noor 2068 (KUH)	Astore, Gilgit-Baltistan, Pakistan	PZ938935	PZ959013	PZ939618	PZ939622	PZ939626

As previous phylogenetic studies of *Melanoseris* have documented widespread topological incongruence between nrITS and plastid datasets (Wang et al. 2013; Kilian et al. 2017; Yin et al. 2018), we analysed these as separate matrices. Sequence alignment of nrITS and the four plastid loci was conducted following the alignment strategies described in Wang et al. (2013). All phylogenetic reconstructions were performed in PhyloSuite v1.2.331 (Xiang et al. 2023). Optimal nucleotide substitution models were screened via IQ-TREE’s ModelFinder based on the Akaike Information Criterion (AIC). Maximum Likelihood (ML) trees were inferred in IQ-TREE with 1,000 standard bootstrap replicates for branch support estimation, while Bayesian Inference (BI) trees were generated in MrBayes with 2,000,000 Markov chain Monte Carlo generations with default parameters. Final phylogenetic trees were visualized and annotated in FigTree v.1.4.4.

## Results

### Morphological and micromorphological characters

Morphological comparisons identified a suite of stable diagnostic features separating *Melanoseris zhufengensis* sp. nov. from *M. violifolia* and *M. rapunculoides*. The new species is caespitose, with stems profusely branched from the base and sessile middle cauline leaves bearing broad, unconstricted basal wings. By contrast, the two allied species have solitary stems and petiolate middle cauline leaves with constricted leaf-base wings. The phyllaries of this new ta­xon are abaxially moderately white-pilose along the midvein, whereas those of the related taxa are glabrous or subglabrous. Furthermore, *M. zhufengensis* sp. nov. produces longer achenes (9.5–10.6 mm) with strongly broadened lateral ribs and a distinct beak (3.1–3.8 mm). Full morphological comparisons with close relatives are summarized in Table 2.

**Table 2.** Morphological comparisons between *Melanoseris zhufengensis*, *M. rapunculoides*, and *M. violifolia*.

**Characters**	***M. zhufengensis***	***M. rapunculoides***	***M. violifolia***
**Stem**	caespitose, profusely branched from base	solitary, paniculately branched apically	solitary, branched only apically
**Basal / lower leaves**	petiole entirely winged, 0.7–3.9 cm; blade lyrately pinnatipartite, lateral lobes 1–5 pairs, glabrous	petiole narrowly winged, 10–15 cm; blade undivided or pinnatifid, sparsely puberulous to subhirsute	petiole winged only toward base, 5–6 cm; blade undivided or lyrately pinnatisect, lateral lobes 0–1 pair, glabrous
**Middle cauline leaves**	sessile, petiolar wing broadly expanded and unconstricted at blade base	petiolate, wing distinctly constricted at blade base	petiolate, wing distinctly narrowed toward blade base
**Phyllaries**	abaxial midvein moderately white-pilose	abaxial midvein glabrous or subglabrous	glabrous
**Achene length**	9.5–10.6 mm	6.5–9 mm	6–7 mm
**Achene ribs**	3 main ribs/face, lateral ones markedly broadened	5–7 main ribs/face, lateral ones weakly broadened	3 main ribs/face, lateral ones weakly broadened
**Achene epidermal sculpturing**	smooth	papillose	papillose
**Achene beak**	present, 3.1–3.8 mm long	present, 1.5–3 mm long	absent, apex truncate

**Figure 1.**
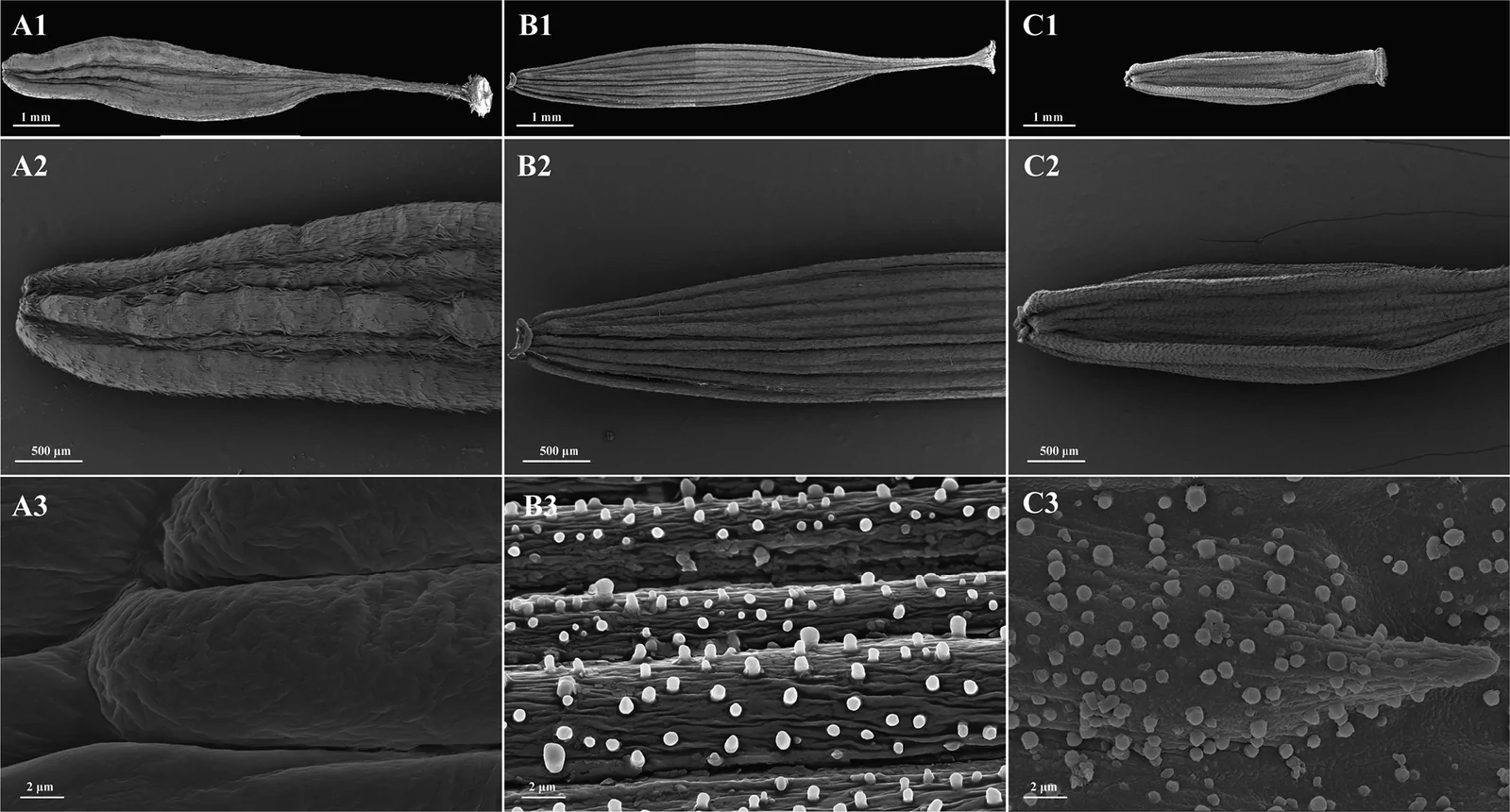
Achene morphology of *Melanoseris zhufengensis* Ze H.Wang & Yi Yang (**A1–A3**), *M. rapunculoides* (D.Don) N.Kilian & Ze H.Wang (**B1–B3**), and *M. violifolia* (Franch.) N.Kilian & Ze H.Wang (**C1–C3**). **A1, B1, C1**. Stereomicrographs of intact achenes; **A2, B2, C2**. SEM micrographs of achene surface; **A3, B3, C3**. Close-up of achene epidermal sculpturing.

In addition to gross morphological divergence, micromorphological observations of achenes using scanning electron microscopy reveal consistent interspecific differences in epidermal sculpturing among these taxa. The achene epidermis of *Melanoseris zhufengensis* sp. nov. is smooth, while its sympatric allies *M. violifolia* and *M. rapunculoides* exhibit papillate epidermal surfaces and lack prominently broadened lateral ribs (Fig. 1). Such micromorphological traits provide complementary evidence for distinguishing the new species from its close relatives.

### Molecular phylogenetic relationships

Two independent alignments were analysed: an nrITS matrix and a combined matrix of four plastid loci (*petD*, *trnL-F*, *rpl32-trnL*, *trnQ-rps16*). Both Bayesian Inference (BI) and Maximum Likelihood (ML) reconstructions were performed for each dataset. The nrITS alignment contained 47 accessions across 654 aligned columns, with 145 parsimony-informative sites. ModelFinder selected GTR+G4 as the optimal nucleotide substitution model under the Akaike Information Criterion (AIC) for the unpartitioned alignment, and it was applied in both BI and ML analyses. For the combined plastid matrix, the dataset included 41 taxa spanning 3,788 aligned columns and contained 146 parsimony-informative sites. Edge-unlinked partitioned model selection via IQ-TREE’s ModelFinder under the AIC was performed twice with constrained candidate model pools matching the respective phylogenetic software and returned distinct best-fit models for BI and ML: all plastid partitions used GTR+F+G4 for BI reconstruction (restricted to MrBayes-compatible models), whereas TVM+F+G4 was chosen for ML infe­rence (full model set for IQ-TREE).

**Figure 2.**
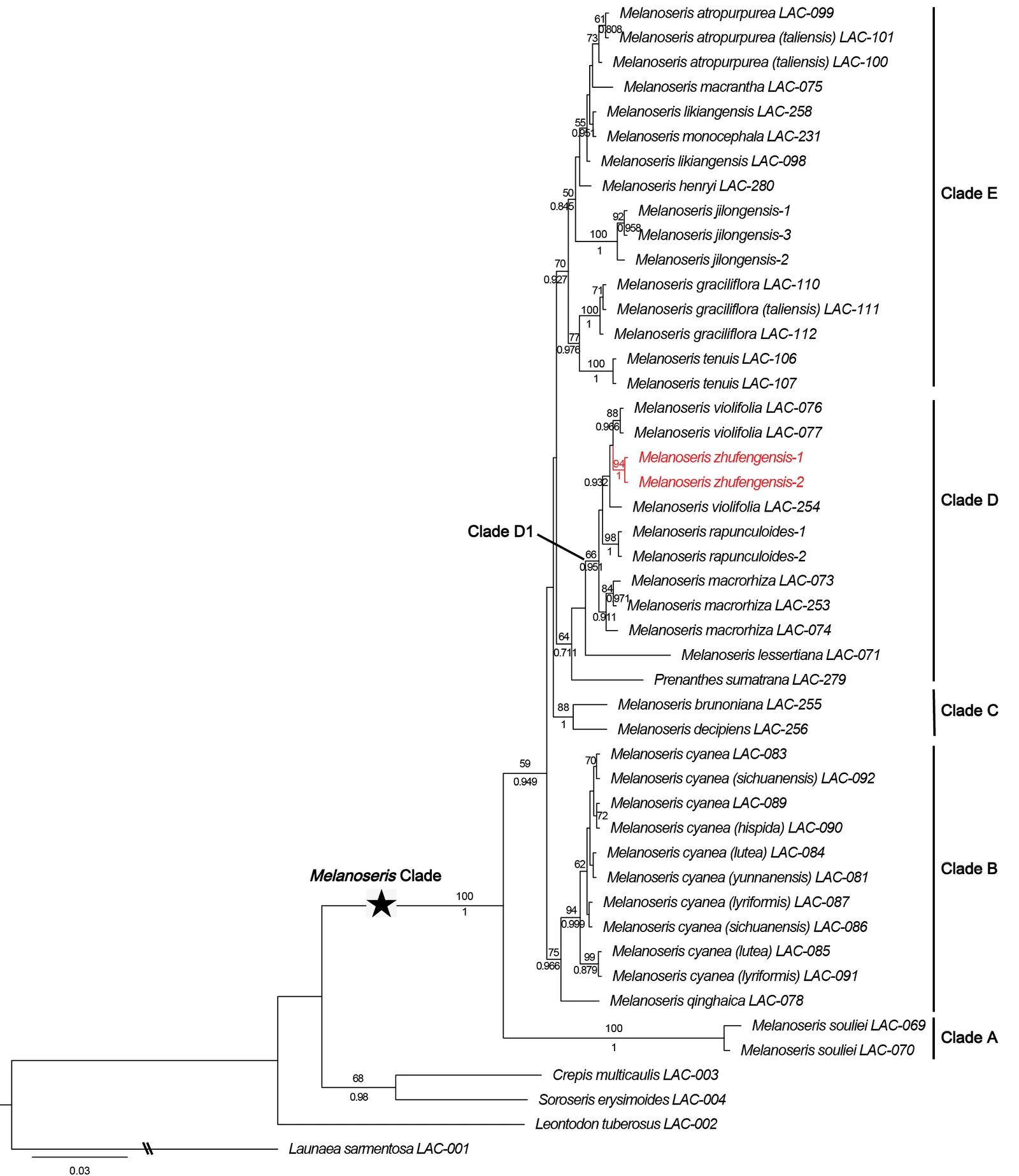
Bayesian consensus tree of *Melanoseris* inferred from the nrITS region. ML bootstrap support (BS) values are shown above branches, and Bayesian posterior probabilities (PP) are indicated below branches.

**Figure 3.**
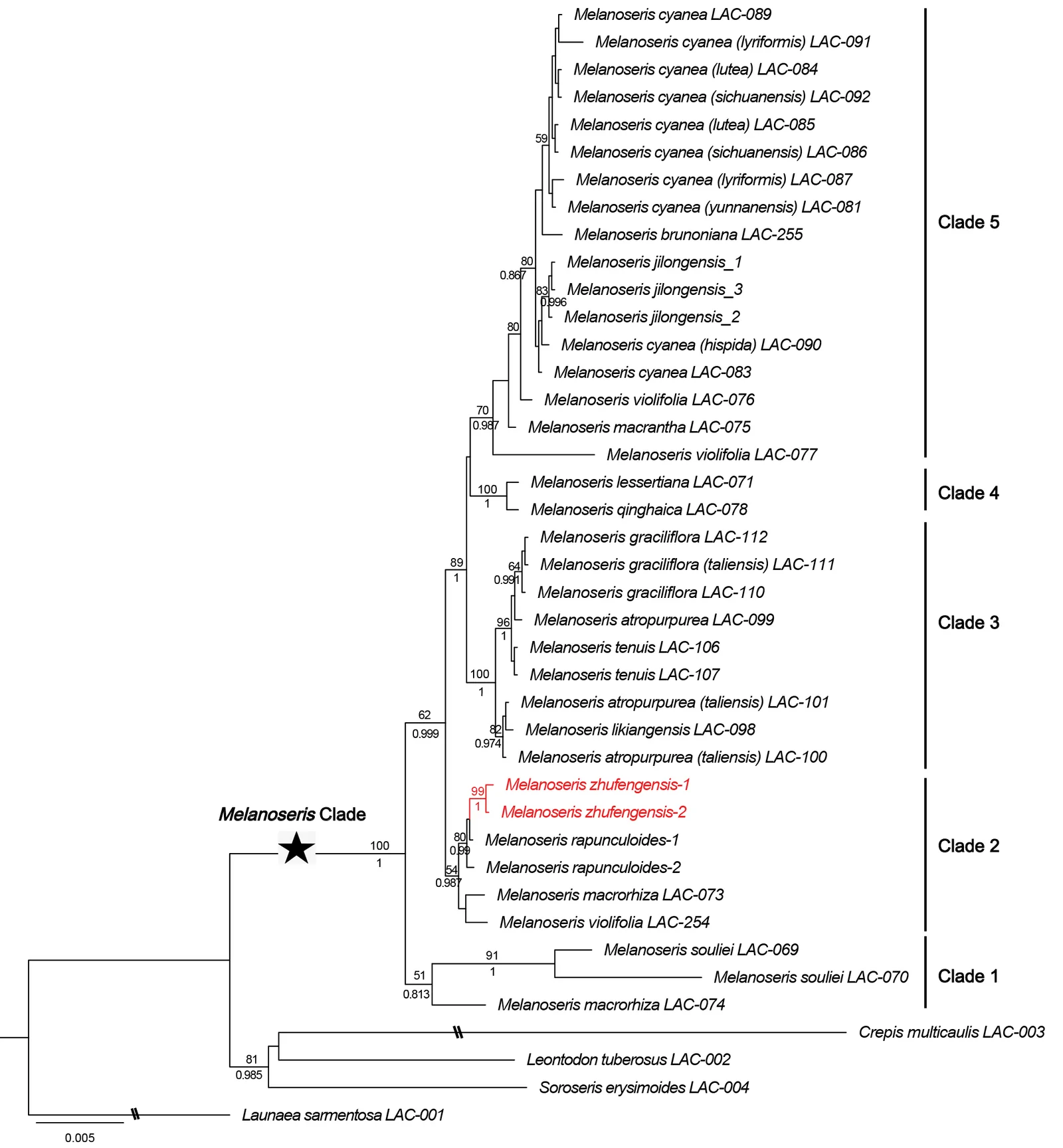
Bayesian consensus tree of *Melanoseris* inferred from concatenated sequences of four plastid loci (*petD*, *trnL-F*, *rpl32-trnL*, *trnQ-rps16*). ML bootstrap support (BS) values are shown above branches, and Bayesian posterior probabilities (PP) are indicated below branches.

ML topologies were highly congruent with their corresponding BI trees; se­parate ML phylograms are not provided, but ML bootstrap support (BS) values are annotated on each BI consensus tree. The nrITS-derived BI tree is presen­ted as Fig. 2, while the plastid BI tree is shown as Fig. 3. Both BI phylogenies recovered the genus *Melanoseris* as a fully supported monophyletic clade (BS = 100, PP = 1.00). The nrITS tree resolved five well-defined major infra-generic lineages (Clade A–E), largely consistent with the subdivision framework proposed in prior taxonomic studies (Wang et al. 2013; Kilian et al. 2017; Yin et al. 2018). The plastid tree also recovered five principal clades (Clade 1–5); separate naming schemes were used for clades from the two datasets to eliminate confusion during inter-tree comparison. Widespread topological incongruences were observed between the nrITS and plastid phylogenies, including discrepancies in deep clade arrangement and internal branching order for many taxa, yet the phylogenetic position of the new species remained largely consistent across both datasets.

Both accessions of the new species *Melanoseris zhufengensis* sp. nov. formed a robust monophyletic lineage. This lineage received strong in both reconstructions: BS = 94, PP = 1.00 in the nrITS tree (Fig. 2), and BS = 99, PP = 1.00 in the plastid tree (Fig. 3). This monophyletic group nested within Clade D in the nrITS topology (BS = 64, PP = 0.711) and fell into Clade 2 in the plastid tree (BS = 54, PP = 0.987). The sister relationship of *M. zhufengensis* sp. nov. varied slightly between datasets: the new species was resolved as sister to the clade of two *M. violifolia* accessions (LAC-076, LAC-077) in the nrITS tree, whereas in the plastid phylogeny only *M. violifolia* LAC-254 fell within this subclade, and *M. zhufengensis* sp. nov. formed a sister pair with *M. rapunculoides*. Furthermore, *M. macrorhiza* was also associated with this subclade but exhibited variable placement of its accessions between datasets. However, representatives of these four taxa consistently formed a tight subclade across the analyses, though the accessions included for *M. violifolia* and *M. macrorhiza* differed between trees (Clade D1: BS = 66, PP = 0.951, Fig. 2; Clade 2: BS = 54, PP = 0.987, Fig. 3), confirming their close phylogenetic affinity.

### Taxonomy

#### Melanoseris zhufengensis

Taxon classificationPlantaeCeramialesRhodomelaceae

Ze H.Wang & Yi Yang sp. nov.

05B23C8E-3091-56D2-A45F-CACDE6001C88

urn:lsid:ipni.org:names:77471301-1

Figs 4, 5

##### Type.

China • Xizang Autonomous Region, Shigatse City, Tingri County, Qudang Town, 28°06'43.8"N, 87°20'29.4"E, alt. 3,610 m a.s.l., 14 Aug 2023, *Ye Chunxia 20230801* (holotype: KUN!, isotypes: GZTM!, LBG!).

**Figure 4.**
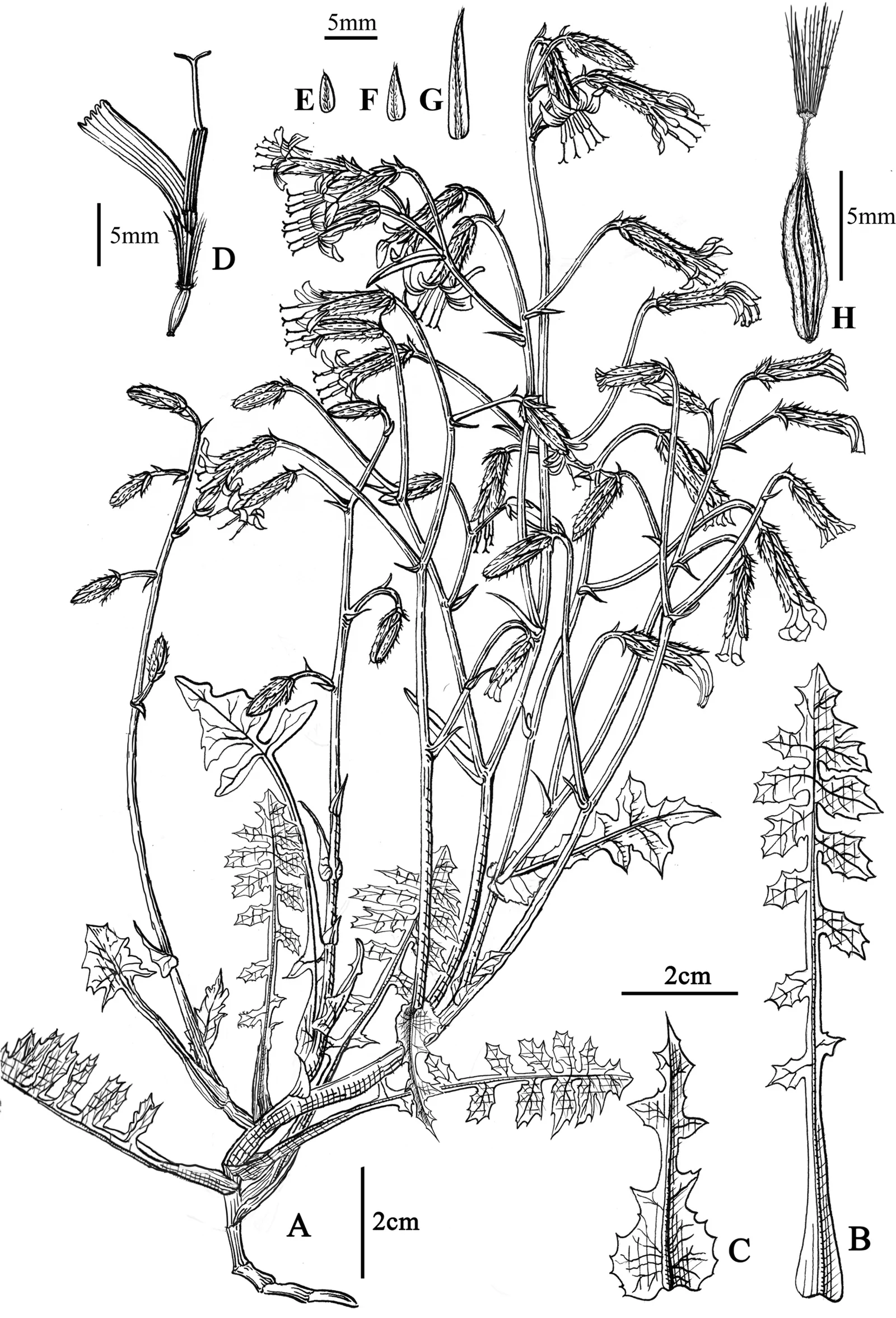
*Melanoseris zhufengensis* Ze H.Wang & Yi Yang, sp. nov. **A**. Habit; **B**. Basal leaf; **C**. Cauline leaf; **D**. Ligulate floret; **E–G**. Phyllaries: outer (**E**), middle (**F**), inner (**G**); **H**. Achene. Drawn by Xiangli Wu.

**Figure 5.**
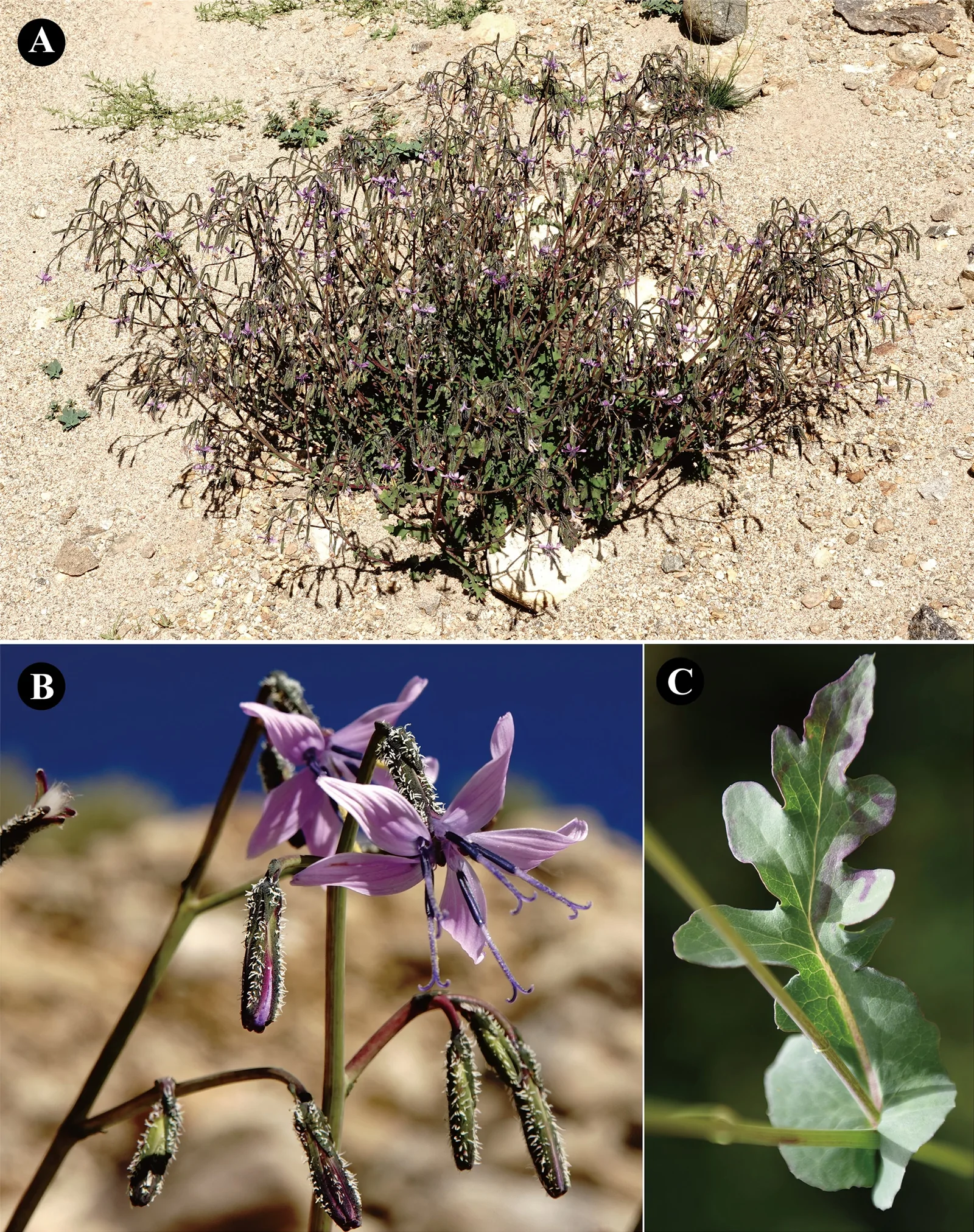
Photographs of *Melanoseris zhufengensis* Ze H.Wang & Yi Yang, sp. nov. **A**. Habit; **B**. Capitula and florets; **C**. Cauline leaf. (**A**) and (**B**) Photographed by Chun-Xia Ye, C Photographed by Guo-Xing Peng.

##### Diagnosis.

*Melanoseris zhufengensis* is similar to *M. violifolia* and *M. rapunculoides* in bearing five florets per capitulum, but readily distinguished by its caespitose habit, stems profusely branched from the base, middle cauline leaves sessile with broad basal wings unconstricted at the blade base, phyllaries abaxially moderately white-pilose along the midvein, and achenes with three markedly broadened lateral ribs on each surface and bearing a slender beak 3.1–3.8 mm long.

##### Description.

Perennial herbs, 20–26 cm tall. Root not seen. Stems profusely branched from the base, erect or ascending, green or reddish brown, glabrous or sparsely pubescent apically. Leaves simple, dimorphic. Basal and lower cauline leaves petiolate, petioles entirely narrowly winged throughout or only expanded at base, 0.7–3.9 cm long, glabrous or sparsely pubescent; blades elongated-oblong, 4–11 × 2–2.5 cm, lyrately pinnatipartite, terminal lobe triangular or broadly triangular, 1.2–1.9 × 1.2–2.1 cm, apex acute; lateral lobes 1–5 pairs, triangular or oblong, 0.5–1.2 × 0.3–0.5 cm, apex obtuse or acute, margins irregularly crenate-denticulate, glabrous on both surfaces. Middle cauline leaves sessile, base broadly winged and auriculately clasping the stem; blade obovate-oblong, pinnately lobed; terminal lobe broadly elliptic to suborbicular, 2.3–5.0 × 0.6–2.0 cm; lateral lobes 1–3 pairs, triangular, 0.5–0.9 × 0.4–0.7 cm, apex obtuse or acute, margins entire or sparsely denticulate, glabrous on both surfaces. Uppermost cauline leaves reduced, lanceolate to broadly linear, entire, base auriculately clasping the stem, becoming bracteiform on synflorescence branches. Synflorescence very lax, diffusely paniculately corymbose, branches slender and flexuous. Peduncles slender, 0.8–4.1 cm long, glabrous or sparsely pubescent. Capitula homogamous, liguliflorous, with 5 florets; involucre cylindrical to narrowly campanulate, 1.2–1.5 × 0.2–0.3 cm. Phyllaries green to dark purplish-green, midvein abaxially moderately white-pilose; outer phyllaries triangular-ovate to lanceolate, the longest ca. 1/2 the length of inner phyllaries; innermost phyllaries 5, linear-lanceolate. Ligules purplish-pink to pale purple, ca. 1.8 cm long. Stamens dark purple; anther tube ca. 0.7 cm long. Ovary ellipsoid, ca. 0.4 cm long; style exserted, ca. 1.9 cm long. Achene 9.5–10.6 mm long; body dark brown, ellipsoid-fusiform, compressed, lateral ribs markedly broadened, 3 ribs on each surface, scabrid-hispid, attenuate apically into a pale beak 3.1–3.8 mm long. Pappus white, dimorphic; outer series of minute hairs 0.1–0.2 mm long; inner bristles scabrid, ca. 6–7 mm long.

##### Distribution and habitat.

*Melanoseris zhufengensis* is currently known only from Qudang Town, Tingri County, Shigatse City, Xizang Autonomous Region, China, where it occurs at elevations ranging from ca. 3,600 m to 3,650 m a.s.l., inhabiting alpine gravelly slopes and alpine meadows.

##### Phenology.

Flowering and fruiting from July to September.

##### Etymology.

The specific epithet ‘zhufengensis’ refers to Mount Zhufeng (Mount Qomolangma), as the type locality of this new species lies in Qomolangma National Nature Reserve within the Central Himalaya of Xizang, China.

##### Vernacular name.

Simplified Chinese: 珠峰毛鳞菊; Chinese Pinyin: Zhūfēng Máolínjú.

##### Additional specimens examined.

China • Xizang Autonomous Region, Shigatse City, Tingri County, Qudang Town, Renqinglin Village, 28°07.62'N, 087°20.65'E, alt. 3,630 m a.s.l., 11 Jul 2024, *Tang Ming et al. JXAU-36* (GTZM!).

## Discussion

Our multi-locus phylogenetic analyses based on nrITS and four plastid markers resolve *Melanoseris zhufengensis* sp. nov. within a weakly to moderately supported subclade together with *M. violifolia*, *M. rapunculoides*, and *M. macrorhiza* in both the nrITS and plastid trees (nrITS: BS = 66, PP = 0.951, Fig. 2; plastid: BS = 54, PP = 0.987, Fig. 3). These four Himalayan endemic taxa share a consistent suite of vegetative and floral phenotypic features, including relatively short plant stature, small floret numbers per capitulum, and basally expanded, auriculate-clasping leaf bases, which morphologically corroborates their close phylogenetic affinity recovered from the nuclear phylogeny.

The vegetative and floral similarity among the constituent species of the subclade inevitably complicates morphological species delimitation. Notably, topological discordance was detected between nuclear and plastid datasets within this species group: although the four taxa form a coherent subclade in the nrITS phylogeny, some plastid accessions of *M. violifolia* and *M. macrorhiza* are instead either placed with the *M. cyanea* clade or with the isolated *M. souliei* clade. Such incongruence is most plausibly attributed to reticulate evolution and chloroplast capture. Natural hybridization and cytoplasmic introgression, the major causes of reticulate phylogenetic patterns, are widespread within Himalayan *Melanoseris* and have been well documented in previous systematic studies, as exemplified by the putative hybrid ori­gin of *M. jilongensis* (Wang et al. 2013; Kilian et al. 2017; Yin et al. 2018). These evolutionary processes frequently obscure strictly bifurcating lineage divergence. Coupled with the strong convergent selection pressure in alpine habitats, closely related species retain highly conserved vegetative and floral morphology, further increasing the difficulty of morphological discrimination.

In contrast to plastic vegetative and floral features, macro- and micromorphological characters of the achenes have been proven to be evolutionarily conservative and taxonomically diagnostic throughout the tribe Cichorieae, providing reliable evidence for delimiting morphologically similar species (Zhu et al. 2006; Abid and Qaiser 2015; Liu et al. 2015). Previous studies on *Melanoseris* have also demonstrated that even morphologically similar species can be reliably distinguished by distinct achene microsculpturing; for instance, *M. jilongensis* is morphologically similar to *M. macrorhiza* in vegetative features but differs substantially in achene micromorphology (Yin et al. 2018). Consistent with these previous findings, the achene micromorphological divergence observed in our study further supports the status of *M. zhufengensis* sp. nov. as a distinct species, reinforcing that integrative molecular data and conservative achene micromorphology constitute a robust framework for resolving cryptic species boundaries within morpholo­gically homogeneous Himalayan *Melanoseris*.

## Supplementary Material

XML Treatment for Melanoseris zhufengensis

## Additional information

### Artificial Intelligence (AI) use

The authors accept full responsibility for the content of the manuscript, including the disclosure of any use of AI.

Regarding the use of AI in the preparation of this manuscript, the authors declare the following: Doubao AI (developed by ByteDance).

**Used for**: Language, style and writing.

Doubao AI (ByteDance) was used only for language polishing, grammar correction and phrasing revision of the manuscript. All morphological observations, molecular data analyses, research design, taxonomic conclusions and core scientific contents were independently performed, reviewed and verified by the authors.

### Author ORCIDs

L. Yan https://orcid.org/0009-0002-6031-7280

Y. Yang https://orcid.org/0000-0003-1996-5446

M. Tang https://orcid.org/0000-0001-8419-0996

Q.-W. Sun https://orcid.org/0000-0003-0848-5017

Z.-H. Wang https://orcid.org/0000-0002-7369-5005

### Data availability

All data that support the findings of this study are available on GenBank, and in the main text.
